# Morphological analysis of peritoneal fluid in fumarate hydratase deficiency-associated renal cell carcinoma: a case report

**DOI:** 10.3389/fonc.2025.1586283

**Published:** 2025-07-03

**Authors:** Yingying Du, Yu Shang, Chang Shi

**Affiliations:** Department of Pathology, The First Affiliated Hospital of Dalian Medical University, Dalian, Liaoning, China

**Keywords:** peritoneal effusion, fumarate hydratase deficiency, renal cell carcinoma, cytological diagnosis, cell block (CB)

## Abstract

Fumarate hydratase deficiency-associated renal cell carcinoma (FH-RCC) is a rare, aggressive subtype of renal cell carcinoma (RCC) driven by FH gene mutations. Its non-specific imaging features and heterogeneous pathological morphology complicate early diagnosis. This case report describes the cytological evaluation of peritoneal fluid from a 35-year-old male with FH-RCC, highlighting distinct cellular features within the effusion. These findings provide insights into the clinicopathological differential diagnosis of serous cavity effusions, potentially enhancing the recognition and management of this uncommon malignancy.

## 1 Introduction

Fumarate hydratase deficiency-associated renal cell carcinoma (FH-RCC) is an infrequent subtype of renal cell carcinoma (RCC) resulting from germline or somatic mutations in the FH gene (1). Germline mutations are linked to hereditary leiomyomatosis and renal cell carcinoma syndrome (HLRCC), an autosomal dominant condition characterized by renal malignancies, uterine leiomyomas, and cutaneous leiomyomas (2). Compared to common RCC subtypes, such as clear cell RCC (ccRCC), FH-RCC exhibits earlier onset, aggressive behavior, and a propensity for early metastasis (1). However, its rarity limits comprehensive understanding of its multi-omics profile (3), including genomics, epigenomics, and immune microenvironment in metastatic settings. Non-specific imaging and clinical presentations further challenge timely diagnosis. This report presents a cytological analysis of peritoneal fluid in a patient with FH-RCC, aiming to delineate characteristic morphological features that may facilitate early detection and inform clinical management.

## 2 Case presentation

A 35-year-old male presented with significant peritoneal effusion, thoracolumbar vertebral destruction, and hypodense liver lesions on imaging. Twenty-two months prior, he underwent radical nephrectomy for a renal tumor, with postoperative pathology confirming HLRCC-associated FH-RCC. Post-surgery, he received tiragolumab and cabozantinib combination therapy. Subsequent bone and lung metastases prompted treatment with bevacizumab, lenvatinib, and toripalimab. Six months later, massive peritoneal effusion developed, necessitating aspiration. Liquid-based cytology and cell block sections were analyzed. This study was approved by the Ethics Committee of the First Affiliated Hospital of Dalian Medical University.

**Figure 1:**
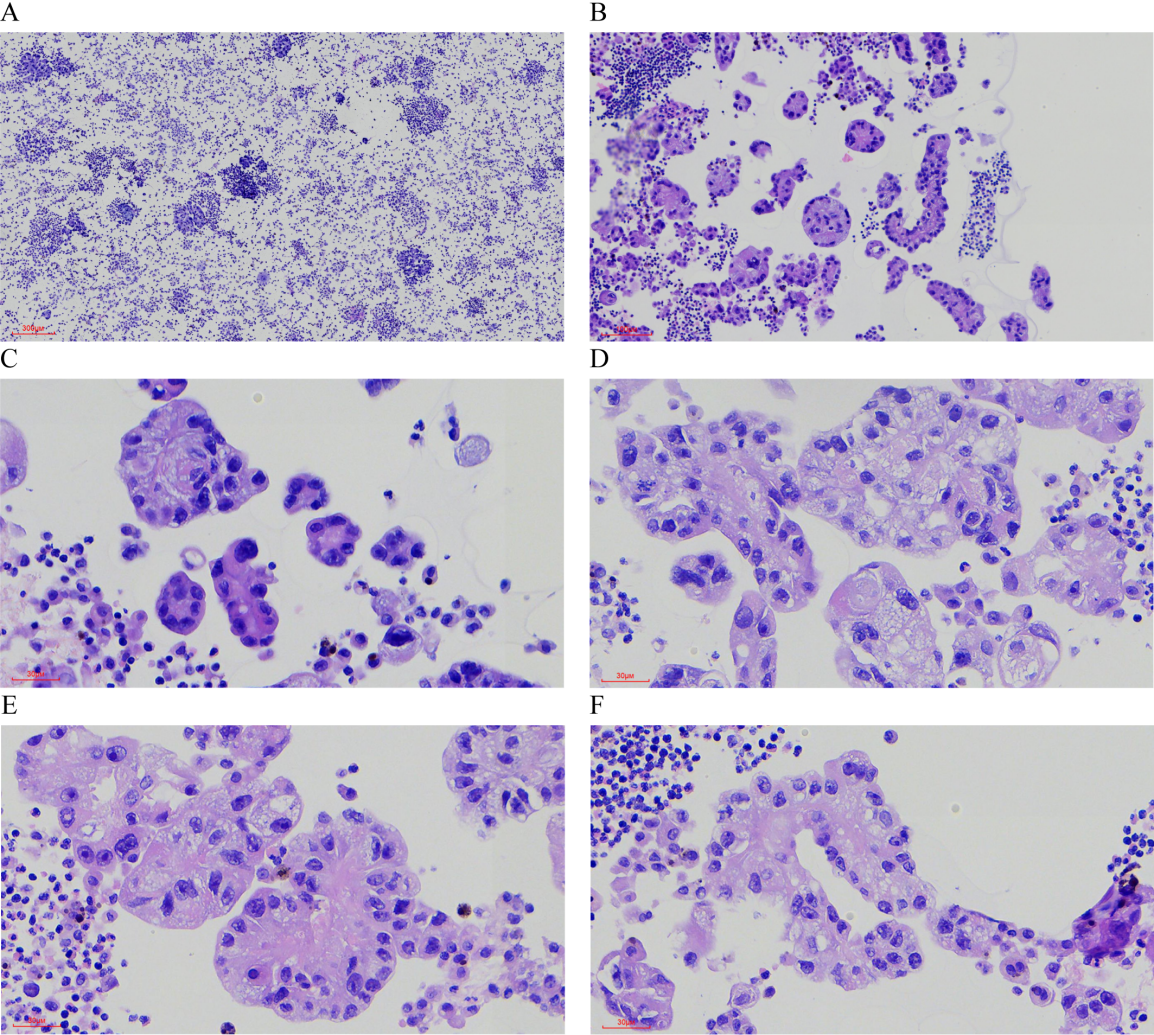
Hematoxylin-Eosin (HE) Staining of Tumor Cells in Ascitic Fluid. **(A)** Tumor cells are numerous and densely packed in the ascitic fluid. (×40) **(B)** The tumor cells exhibit a variety of growth patterns, including papillary, solid, and nested structures. (×100) **(C-F)** The cytoplasm is abundant, eosinophilic or transparent, with some cells showing amphophilic staining or cytoplasmic vacuoles. (×400) **(E, F)** Prominent eosinophilic nucleoli are visible, some surrounded by a distinct halo (×400).

**Figure 2:**
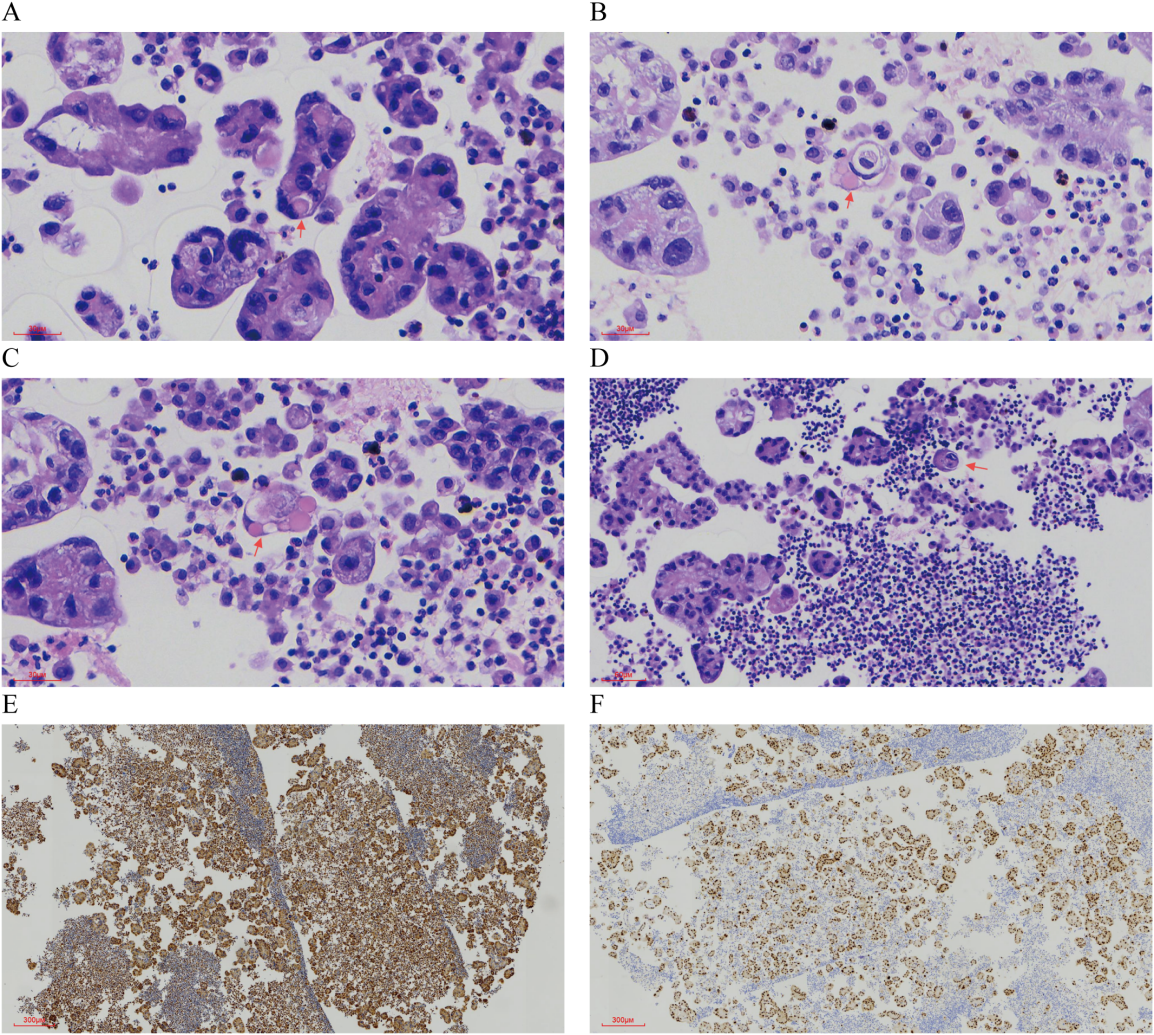
Characteristic Morphology and Immunohistochemical Features in Ascitic Fluid. **(A-C)** Eosinophilic spherules are observed. (×400) **(D)** Tumor cells exhibit phagocytosis. (×200) **(E)** Immunohistochemical staining for FH is positive. (×40) **(F)** Immunohistochemical staining for PAX8 is positive (×40).

**Figure 3:**
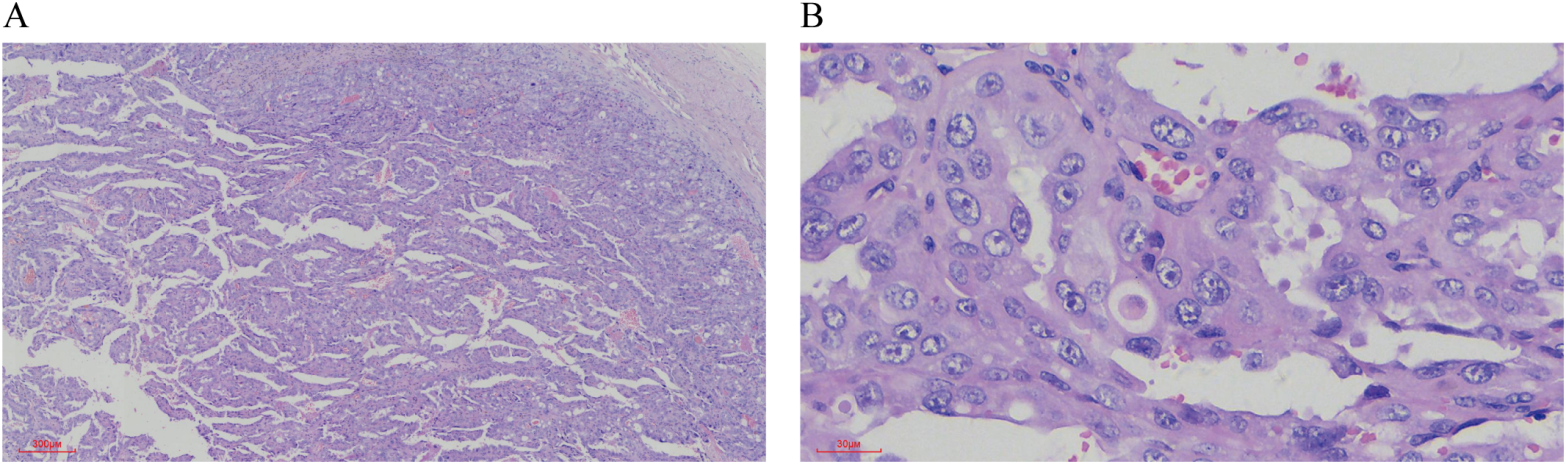
HE staining of Postoperative Renal Tumor Features. **(A)** The tumor tissue displays a mixture of papillary, nested, and solid growth patterns. (×40) **(B)** The tumor cells are large, with either eosinophilic or vacuolated cytoplasm. The nuclei vary in size, and prominent eosinophilic nucleoli are evident (×400).

Pathological Findings: Liquid-based cytology revealed abundant tumor cells arranged in papillary formations, with areas of solid, nested, or dispersed single cells (**Figures 1A, B**). Tumor cells were large, with indistinct borders, eosinophilic or clear cytoplasm, and occasional biphasic staining. Cytoplasmic vacuoles were observed in some cells (**Figures 1C–F**). Nuclei displayed variable size and shape, irregular contours, prominent eosinophilic nucleoli, and occasional perinucleolar halos (**Figures 1E, F**). Phagocytosis of similar cells and eosinophilic globular material was noted in some tumor cells (**Figures 2A–D**). Immunophenotype (Peritoneal Fluid): 2SC (+), Ber-EP4 (+), FH (+), CK (+), Ki-67 (30% in hotspots), MOC-31 (+), Pax-8 (+), P53 (wild-type), CAIX (+), CR (-), Desmin (-), WT-1 (-), CK20 (-), CK7 (-), P40 (-) (**Figures 2E, F**). Post-Nephrectomy Pathology: The primary tumor exhibited papillary, nested, and solid architecture with prominent nucleoli (**Figures 3A, B**). Tumor emboli were identified in blood vessels and the renal vein. Immunophenotype (Primary Tumor): 2SC (+), CAIX (+), CD10 (-), CK (+), EMA (+), FH (+), Ki-67 (30% in hotspots), TFE3 (-), CK20 (-). Diagnosis: Fumarate hydratase-deficient renal cell carcinoma (FH-RCC).

Treatment Course: Three months post-nephrectomy, the patient began tiragolumab and cabozantinib. One year later, bone and lung metastases emerged, leading to a regimen of bevacizumab, lenvatinib, and toripalimab. Six months into this treatment, significant peritoneal effusion developed.

## 3 Discussion

Fumarate hydratase-deficient renal cell carcinoma (FH-RCC), first described by Launonen et al. in 2001, arises from FH gene mutations or inactivation on chromosome 1q42.3-43 (2, 4). This leads to fumarate accumulation, stabilizing hypoxia-inducible factor 1-alpha (HIF1α) and driving metabolic dysregulation and tumorigenesis. Classified under HLRCC in the 2016 WHO renal tumor classification (4th edition), FH-RCC was redefined in the 2022 WHO update to encompass all RCCs with FH alterations (5, 6). According to the 5th edition of the WHO classification, the required diagnostic criteria for fumarate hydratase-deficient renal cell carcinoma (FH-RCC) include immunohistochemical evidence of FH germline or somatic mutation, or FH deficiency, defined by loss of FH protein expression and/or positive 2SC staining. The supportive diagnostic features include the presence of multiple, admixed architectural patterns with at least focal macronucleoli, and a personal or family history of cutaneous and uterine leiomyomas, which may indicate an association with hereditary leiomyomatosis and renal cell carcinoma (HLRCC) syndrome (7).

FH-RCC typically affects younger patients and presents as a solitary renal mass with non-specific symptoms (8). Its aggressive nature predisposes to early metastasis, commonly to lymph nodes, bones, liver, and lungs (9). In this case, a 35-year-old male developed bone and lung metastases 15 months post-nephrectomy, followed by peritoneal effusion and liver metastasis at 22 months.

Cytologically, FH-RCC is characterized by prominent eosinophilic nucleoli with perinucleolar halos (8). In this patient’ s peritoneal effusion, eosinophilic globular material was also observed, potentially reflecting fibrin or peroxisomal complexes linked to autophagic defects, as suggested by prior studies (10). Similar globules have been reported in papillary and clear cell RCC subtypes, distinguishable by their distinct cytoplasmic boundaries (11, 12).

A review of FH-RCC cases with eosinophilic globules (**Table 1**) revealed: (1) no gender predilection (female: male= 4:3); (2) mean onset age of 38 years (range: 11–50); (3) frequent FH mutations (e.g., G354R, p.K80fs); (4) association with HLRCC features in some cases; (5) variable tumor sizes (0.2–21 cm) and growth patterns (solid, tubular, cystic); (6) consistent FH negativity and 2SC positivity; and (7) variable outcomes, with some patients disease-free post-surgery and others experiencing recurrence or metastasis.

**Table 1:** Summary of clinical and pathological features of cases with eosinophilic globules in fumarate hydratase-deficient renal cell carcinoma.

No.	Publication Date/Author	Gender/Age	HLRCC evidence and family history	Surgical approach	Tumor size(cm)	Pathological morphological features	Immunohistochemistry		Other findings	Follow-up
							FH	2SC		
1	2017/Steven C Smith	Male/11	Germline G354R FH mutation	Radical nephrectomy	8.5	Solid, tubular, and cystic; Eosinophilic cytoplasm; Eosinophilic cytoplasmic inclusions	–	+	/	NED, >84 months
2		Male/40	Homozygous p.K80 fs FH mutation in separate tumours	Radical nephrectomy	0.2 and 0.4	Solid, nodular; Eosinophilic cytoplasm; Eosinophilic cytoplasmic inclusions	–	+	/	Liver metastasis, of high grade, at presentation
3		Male/41	/	Partial nephrectomy	5	Solid; Eosinophilic cytoplasm; Eosinophilic cytoplasmic inclusions	–	+	Renal: Multilocular cystic change	NED, 48 months (oncocytic RCC); metastasis at 3 months after high-grade FHRCC nephrectomy
4	2020/Nicolas Wyvekens	Female/50	Cutaneous leiomyomata; Uterine leiomyoma	Nephrectomy with lymphadenectomy	FHRCC:11 Uterine leiomyoma: none	FHRCC: Tubulocystic and papillary growth pattern, prominent nucleoli, perinucleolar halos; Uterine leiomyoma: Staghorn-like blood vessels, high cellularity, ovoid nuclei, prominent nucleoli, eosinophilic cytoplasmic globules	FHRCC: -; Uterine leiomyoma: -	/	/	Local recurrence and subsequent treatment one year later; NED, 14 months
5		Female/46	Cutaneous leiomyomata; Uterine leiomyoma	Partial nephrectomy	First recurrent tumor nodule:1.2 Second local recurrence:1.3-3(multiple retroperitoneal nodules) Lymph node metastases:4 Uterine leiomyoma: none	FHRCC recurrence and metastasis: Papillary growth pattern, large tumor cells, prominent nucleoli with perinuclear halos, abundant cytoplasmic “colloid-like” inclusions; Uterine leiomyoma: Alveolar edema, staghorn-like blood vessels, intracytoplasmic globules	FHRCC recurrence: -; Uterine leiomyoma: -	/	/	Local recurrence
6	2022/Athanase Billis	Female/45	Uterine leiomyoma	/	7.5	Papillary arrangement, solid arrangement, tubulocystic arrangement, eosinophilic cytoplasm, large nucleoli surrounded by a clear halo, eosinophilic inclusions (round and oval)	–	+	/	NED, 15 months
7		Female/35	Uterine leiomyoma	/	21	Papillary arrangement, solid arrangement, eosinophilic cytoplasm, large nucleoli surrounded by a clear halo, eosinophilic inclusions (round and oval)	–	+	/	NED, 32 months

Differential diagnosis of peritoneal effusion includes: (1) Ovarian Cancer: Features irregular clusters or glandular structures, often with calcified psammoma bodies in serous carcinoma, contrasting with FH-RCC’s eosinophilic nucleoli and “owl-eye” appearance (13–15). (2) Gastrointestinal Adenocarcinoma: Exhibits glandular or signet-ring morphology with PAS-positive vacuoles, distinguishable by negative CDX-2 and villin in FH-RCC. (3) Mesothelioma: Shows scattered or clustered cells with basophilic cytoplasm and BAP1 loss in ~60% of cases, unlike FH-RCC’s eosinophilic features (16).

The presence of eosinophilic globules in this case, alongside characteristic nucleoli and immunoprofile (FH loss, 2SC positivity), strongly supports FH-RCC diagnosis. Absent FH IHC staining in tumor cells is highly specific for FH-RCC, but its sensitivity is limited. In contrast, diffuse expression of 2-succinocysteine (2SC), resulting from aberrant cysteine modification due to fumarate accumulation, is highly sensitive but lacks specificity. Additionally, a recently described pattern of heterogeneous FH expression—characterized by patchy positive staining—has been associated with FHRCC. This phenomenon is thought to result from certain missense mutations that impair FH enzymatic activity while allowing for variable protein expression (17–21). In this case, immunohistochemical staining showed diffuse and strong positivity for 2SC, whereas FH demonstrated a patchy immunoreactivity pattern.

Therapeutically, FH-RCC lacks standardized guidelines. Bevacizumab with erlotinib has shown partial efficacy in HLRCC-associated RCC, while immune checkpoint inhibitors (ICIs) with anti-angiogenic agents yield objective response rates (ORR) of 27–44% in retrospective studies (22–24). In this patient, despite sequential therapies (tiragolumab/cabozantinib, then bevacizumab/lenvatinib/toripalimab), disease progression persisted, highlighting therapeutic challenges in advanced FH-RCC.

## 4 Conclusion

This report presents the first cytological description of FH-RCC in peritoneal effusion, emphasizing distinctive features—eosinophilic nucleoli, perinucleolar halos, and globular material—that aid differential diagnosis. Despite multimodal therapy, disease progression underscores the aggressive nature and poor prognosis of some FH-RCC cases, necessitating further research into effective treatments.

## Data Availability

The original contributions presented in the study are included in the article/supplementary material. Further inquiries can be directed to the corresponding author.
